# Supplementary material for: Evolution of retinal degeneration and prediction of disease activity in relapsing and progressive multiple sclerosis
Source: Nat Commun. 2024 Jun 19;15:5243. doi: 10.1038/s41467-024-49309-7 (PMC11187157; doi:10.1038/s41467-024-49309-7)
Supplement: Supplementary file 1 — Supplementary Information [file 41467_2024_49309_MOESM1_ESM.pdf]

Supplementary Information

Supplementary Table 1 Number of excluded eyes

**Reasons for exclusion**	**RRMS**	**SPMS**	**PPMS**	**HC**
Insufficient OCT signal	43	8	39	11
Drusen	8	19	17	7
Myopia ≥ ± 6 dpt				1
Amblyopia	3			1
Foveal retinal pigment epithelium lesion	1			
Glaucoma	4	6	6	
Retinal hole	1			
Optic neuritis ≤ 6 months before baseline or during follow-up	30	6		
Macular edema	4	6		2
Retinitis pigmentosa	2			
Transition to SPMS during follow-up	6			
No fullfilled diagnosis criteria	4			
Missing clinical data	24			
Too short follow-up (≤ 9 months)	45	49	54	32
Retinal pathology not further classified	5	6	2	
Epiretinal gliosis	9	5	6	2
Macular dystrophy			2	
Incomplete OCT data		3	8	
OCT impossible due to fixation problems			2	
Traction phenomena		1	2	
Death		2	4	
Normal pressure hydrocephalus			2	
Maculopathy		1	2	
OCT under chemotherapy			2	
Retinal scar			3	
Branch retinal artery occlusion		1		
Macular hole and epiretinal membrane cystic lesion		1		
Bleedings and exudates		2		

CADASIL		2		
Wilson's disease		2		
Head tremor interfering with examination		2		
Foveal pathology		2		
Recurrent uveitis		2	2	
Iridocyclitis			2	
Macular infiltration		1		
Eccentric serous chorioretinopathy	1			
Papillary edema	2			
Amaurosis			1	
Peripapillary retinochisis			1	

Legend: Legend: CADASIL = Cerebral Autosomal Dominant Arteriopathy with Subcortical Infarcts and Leukoencephalopathy; OCT = optical coherence tomography; SPMS = secondary progressive MS. Number of eyes which were excluded from analysis due to different reasons are presented for patients with relapsing-remitting MS (RRMS), secondary progressive MS (SPMS), primary progressive MS (PPMS), and healthy controls (HC) separately.

Supplementary Table 2 P and b values of the linear regressions between retinal layer thickness, visual acuity, and VEP latency at baseline and disease duration when adjusting for optic neuritis, participants' age and/or sex if the model fit could be improved.

	**pRNFL p value**	**pRNFL b value**	**95% CI**	**Covariates**
**RRMS**	**<0.001**	-1.40	-1.96; -0.84	Age, Sex, ON
**PPMS**	0.24	-0.60	-1.61; 0.4	Age, ON
**SPMS**	**<0.001**	-0.78	-1.20; -0.36	Age, Sex, ON
	**mRNFL p value**	**mRNFL b value**		
**RRMS**	**<0.001**	-0.35	-0.54; -0.17	Age, Sex, ON
**PPMS**	0.87	-0.03	-0.35; 0.30	Age, Sex, ON
**SPMS**	**0.002**	-0.17	-0.27; -0.07	Age, Sex, ON
	**GCIPL p value**	**GCIPL b value**		

**RRMS**	**<0.001**	-0.71	-1.07; -0.34	ON
**PPMS**	0.66	-0.15	-0.84; 0.53	Age, ON
**SPMS**	**0.002**	-0.42	-0.64; -0.19	ON
	**INL p value**	**INL b value**		
**RRMS**	0.65	0.03	-0.09; 0.14	Sex, ON
**PPMS**	0.79	0.02	-0.15; 0.20	Age
**SPMS**	0.11	0.07	-0.01; 0.15	Sex, ON
	**Visual acuity p value**	**Visual acuity b value**		
**RRMS**	0.10	0.008	-0.0009; 0.02	Age
**PPMS**	0.85	-0.0009	-0.01; 0.009	Age
**SPMS**	0.23	0.006	-0.004; 0.02	ON
	**VEP Latency p value**	**VEP Latency b value**		
**RRMS**	**0.001**	1.34	0.73; 1.96	Age, Sex, ON
**PPMS**	0.27	0.69	-0.48; 1.85	Age, Sex, ON
**SPMS**	0.98	0.02	-1.16; 1.19	Age, Sex, ON

Legend: Mixed effects linear model regression analyses. Peripapillary retinal nerve fiber layer (pRNFL), macular retinal nerve fiber layer (mRNFL), ganglion cell-inner plexiform layer (GCIPL), and inner nuclear layer (INL) of patients with relapsing-remitting MS (RRMS), secondary progressive MS (SPMS), and primary progressive MS (PPMS). ON was categorized in the categories a) ON based on medical history, b) ON based on inter-eye GCIPL thickness difference [43], and c) no ON). Source data are provided as a Source Data file.

Supplementary Table 3 Overall annualized thickness change rate over time

	**pRNFL Median**	**pRNFL IQR**
**HC**	-0.13	-0.78 - 0.15
**RRMS**	-0.49	-1.44 - 0.00
**PPMS**	-0.39	-1.33 - 0.00
**SPMS**	-0.18	-1.03 - 0.64
	**mRNFL Median**	**mRNFL IQR**
**HC**	0.00	-0.57 - 0.35
**RRMS**	0.00	-0.67 - 0.38
**PPMS**	-0.07	-0.68 - 0.34
**SPMS**	0.00	-0.35 - 0.35
	**GCIPL Median**	**GCIPL IQR**

**HC**	-0.28	-0.60 - 0.11
**RRMS**	-0.18	-0.68 - 0.23
**PPMS**	-0.33	-0.70 - 0.00
**SPMS**	-0.20	-0.55 - 0.00
	**INL Median**	**INL IQR**
**HC**	0.00	-0.34 - 0.23
**RRMS**	0.00	-0.35 - 0.32
**PPMS**	0.00	-0.35 - 0.31
**SPMS**	0.00	-0.35 - 0.27

Legend: Median and inter-quartile range (IQR) in µm of overall annualized rate of thickness change over time of peripapillary retinal nerve fiber layer (pRNFL), macular retinal nerve fiber layer (mRNFL), ganglion cell-inner plexiform layer (GCIPL), and inner nuclear layer (INL) of healthy controls (HC) and patients with relapsing-remitting MS (RRMS), secondary progressive MS (SPMS), and primary progressive MS (PPMS). Source data are provided as a Source Data file.

Supplementary Table 4 P and b values of the overall annualized thickness change rate over time

	**pRNFL p value**	**pRNFL b value (95% CI)**
**HC**		
**RRMS**	**<0.001**	-0.58 (-0.67; -0.49)
**PPMS**	**<0.001#**	-0.82 (-1.10; -0.54)
**SPMS**	**<0.001**	-0.35 (-0.47; -0.23)
	**mRNFL p value**	**mRNFL b value (95% CI)**
**HC**		
**RRMS**	**<0.001**	-0.10 (-0.14; -0.05)
**PPMS**	**<0.001**	-0.17 (-0.25; -0.10)
**SPMS**	**0.004#**	-0.14 (-0.23; -0.05)
	**GCIPL p value**	**GCIPL b value (95% CI)**
**HC**		
**RRMS**	**<0.001**	-0.22 (-0.27; -0.17)
**PPMS**	**<0.001**	-0.25 (-0.31; -0.19)
**SPMS**	**<0.001**	-0.21 (-0.27; -0.15)
	**INL p value**	**INL b value (95% CI)**
**HC**		
**RRMS**	**0.01##**	-0.03 (-0.05; -0.01)
**PPMS**	0.18	-0.02 (-0.05; 0.01)
**SPMS**	**0.04**	-0.03 (-0.06; -0.00)

Legend: P values are provided for the linear mixed effects model analysis including all raw data points (not change rates). The covariates were entered based on goodness of fit. # age was included in the model; ##sex was included in the model. Only the INL has not significantly changed in PPMS patients over time (p = 0.18). Source data are provided as a Source Data file.

Supplementary Table 5 P and b values of the annualized thickness change rates of the different layers for the different periods of the disease duration

	pRNFL p value	pRNFL b value (95% CI)	mRNFL p value	mRNFL b value (95% CI)	GCIPL p value	GCIPL b value (95% CI)	INL p value	INL b value (95% CI)
**HC**								
**0-3.5 years**	**0.01**	-0.42 (-0.73; -0.11)	**<0.001**	-0.27 (-0.39; 0.14)	0.24#	-0.10 (-0.25; 0.06)	0.15	-0.04 (-0.10; 0.02)
**RRMS**								
**0-3.5 years**	**<0.001**	-0.94 (-1.23; -0.65)	**0.04**	-0.14 (-0.27; -0.01)	**<0.001**	-0.34 (-0.48; -0.21)	0.13##	-0.05 (-0.11; 0.01)
**3.6-5.5 years**	**<0.001**	-0.93 (-1.44; -0.42)	0.48	-0.11 (-0.41; 0.19)	**0.04**	-0.28 (-0.53; -0.02)	0.99##	0.001 (-0.14; 0.15)
**5.6-7.5 years**	**0.004**	1.32 (0.56; 2.13)	0.68#	0.11 (-0.41; 0.62)	0.45	0.21 (-0.35; 0.75)	0.24##	0.11 (-0.08; 0.29)
**7.6-10.5 years**	0.15	-1.15 (-3.65; 0.09)	0.09	-0.76 (-1.76; 0.05)	0.08###	-0.38 (-0.83; 0.01)	0.73	0.06 (-0.27; 0.42)
**10.6-13.5 years**	0.12	-1.32 (-2.80; 0.27)	**0.03**	-0.63 (-1.16; -0.09)	**0.03**	-0.88 (-1.70; -0.13)	0.17##	-0.26 (-0.61; 0.12)
**13.6-16.5 years**	**0.03**	-0.73 (-1.37; -0.05)	0.71	-0.04 (-0.22; 0.17)	0.80	-0.04 (-0.33; 0.25)	0.24	-0.14 (-0,39; 0.11)
**PPMS**								
**0-3.5 years**	**0.005**	-0.85 (-1.37; -0.31)	0.26	-0.27 (-0.78; 0.22)	0.06	-0.52 (-1.05; -0.01)	0.17	-0.17 (-0.42; 0.07)
**3.6-5.5 years**	**0.002**	-1.77 (-2.71; -0.84)	0.12	-0.47 (-1.04; 0.12)	0.62	-0.13 (-0.66; 0.38)	0.45	0.12 (-0.21; 0.45)
**5.6-7.5 years**	**0.04**	-0.86 (-1.65; -0.04)	0.88##	-0.02 (-0.35; 0.31)	**0.03**	-0.32 (-0.58; -0.04)	**0.002**	-0.37 (-0.60; -0.15)
**7.6-10.5 years**	0.38	-0.36 (-1.19; 0.47)	0.31	0.28 (-0.25; 0.81)	**0.02**	-0.37 (-0.65; -0.11)	0.36	-0.08 (-0.23; 0.09)
**10.6-13.5 years**	**0.007#**	-1.08 (-1.83; -0.33)	**0.002**	-0.52 (-0.83; -0.21)	0.34	-0.18 (-0.53; 0.19)	0.08	0.24 (-0.03; 0.50)
**13.6-16.5 years**	0.10	-0.45 (-1.04; 0.06)	**0.002##**	-0.34 (-0.54; -0.15)	0.09	-0.25 (-0.54; 0.03)	0.08	-0.15 (-0.31; 0.01)
**16.6-20.5 years**	0.33	-0.45 (-1.37; 0.42)	0.90	-0.03 (-0.44; 0.35)	**0.001**	-0.46 (-0.73; -0.26)	0.37	0.11 (-0.14; 0.36)

**SPMS**								
**3.5-12.5 years**	**0.01**[###]	-1.42 (-2.39; -0.43)	0.49	0.07 (-0.13; 0.28)	0.12	-0.28 (-0.58; -0.05)	0.10	-0.11 (-0.24; 0.02)
**12.6-16.5 years**	0.28	-0.34 (-0.96; 0.27)	0.95	0.01 (-0.23; 0.24)	0.58	-0.10 (-0.44; 0.24)	0.97[##]	-0.003 (-0.17; 0.15)
**16.6-20.5 years**	0.48	-0.15 (-0.58; 0.28)	0.66	-0.07 (-0.38; 0.23)	0.19[##]	-0.11 (-0.26; 0.05)	**0.01**	0.22 (0.05; 0.39)
**20.6-25.5 years**	0.57	-0.24 (-1.06; 0.58)	0.96	0.01 (-0.25; 0.24)	0.13	-0.23 (-0.51; 0.06)	0.21	0.10 (-0.06; 0.26)
**25.6-30.5 years**	**0.02**[###]	-1.32 (-2.40; 0.19)	**0.01**[###]	-0.49 (-0.85; -0.11)	**0.001**[###]	-1.09 (-1.55; -0.62)	0.91	-0.01 (-0.18; 0.16)
**over 30.6 years**	0.19	-0.14 (-0.33; 0.03)	0.47	0.03 (-0.06; 0.12)	**0.005**	-0.16 (-0.25; -0.07)	**0.003**	-0.14 (-0.22; -0.05)

Legend: Mixed effects linear models were used to calculate the annualized thickness change rates (retinal layer thickness$_{\text{current assessment}}$ - retinal layer thickness$_{\text{previous assessment}}$) / time since last assessment in years) of the different layers for the different periods of the disease duration. The values are presented for patients with relapsing-remitting MS (RRMS), primary progressive MS (PPMS), and secondary progressive MS (SPMS), and for time since baseline OCT in healthy controls (HC). P-values are provided for the mixed linear model analysis including all raw data points (not change rates) and potential covariates (see methods for details on model computation). The covariates were entered based on goodness of fit. [#]age was included in the model; [##]sex was included in the model; [###]age and sex were included in the model. Source data are provided as a Source Data file.

Supplementary Table 6 Number of included eyes for each interval of disease duration by disease subtype

	**pRNFL**	**mRNFL**	**GCIPL**	**INL**
**HC**				
**0-3.5 years**	**87**	**211**	**207**	**211**
**RRMS**				
**0-3.5 years**	**249**	**275**	**275**	**275**
**3.6-5.5 years**	**209**	**224**	**224**	**224**
**5.6-7.5 years**	**132**	**145**	**145**	**145**
**7.6-10.5 years**	**73**	**76**	**76**	**76**
**10.6-13.5 years**	**28**	**31**	**31**	**31**
**13.6-16.5 years**	**29**	**33**	**33**	**33**
**PPMS**				

0-3.5 years	19	27	27	27
3.6-5.5 years	46	60	60	60
5.6-7.5 years	51	68	68	68
7.6-10.5 years	49	64	64	64
10.6-13.5 years	50	68	68	68
13.6-16.5 years	35	45	45	45
16.6-20.5 years	28	31	31	31
SPMS				
3.5-12.5 years	20	30	30	30
12.6-16.5 years	38	42	42	42
16.6-20.5 years	38	40	40	40
20.6-25.5 years	32	37	37	37
25.6-30.5 years	38	47	47	47
over 30.6 years	51	57	57	57

Legend: The number of included eyes is presented for each interval of disease duration for peripapillary retinal nerve fiber layer (pRNFL), macular retinal nerve fiber layer (mRNFL), ganglion cell-inner plexiform layer (GCIPL), and inner nuclear layer (INL) for patients with relapsing-remitting MS (RRMS), primary progressive MS (PPMS), and secondary progressive MS (SPMS), and for healthy controls (HC). The disease duration corresponded to the time since baseline OCT in healthy controls.

Supplementary Table 7. Analysis of variance on empirical Bayes estimates of best linear unbiased predictions of thickness change rate.

	Df	F value	p value
**pRNFL**	2,367	3.65	**0.03**
**mRNFL**	2,404	4.43	**0.01**
**GCIPL**	2,404	0.39	0.68
**INL**	2,404	0.63	0.53

Legend: Df=number of degrees of freedom; pRNF=peripapillary retinal nerve fiber layer; mRNFL=macular retinal nerve fiber layer; GCIPL=ganglion cell-inner plexiform layer; INL=inner nuclear layer. All statistical tests were one-sided. Source data are provided as a Source Data file.

Supplementary Table 8. Analysis of variance on eye wise ordinary least squares of thickness change rate with disease duration as only predictor and aggregrated regression coefficients.

	Df	F value	p value
**pRNFL**	2,186	4.144	**0.02**
**mRNFL**	2,215	4.338	**0.01**
**GCIPL**	2,215	0.385	0.68
**INL**	2,213	0.877	0.42

Legend: Df=number of degrees of freedom; pRNF=peripapillary retinal nerve fiber layer; mRNFL=macular retinal nerve fiber layer; GCIPL=ganglion cell-inner plexiform layer; INL=inner nuclear layer. All statistical tests were one-sided. Source data are provided as a Source Data file.

Supplementary Table 9. Cox proportional hazard regression models for baseline pRNFL, mRNFL, GCIPL, INL thickness tertiles as a predictor of disability worsening, MRI progression/activity, and relapses

	Relapse			EDSS Progression			MRI progression/activity		
	Hazard Ratio (95% CI)	p-value	Prob. of event ($N_{event}/N_{non\text{-}events}$)	Hazard Ratio (95% CI)	p-value	Prob. of event ($N_{event}/N_{non\text{-}events}$)	Hazard Ratio (95% CI)	p-value	Prob. of event ($N_{event}/N_{non\text{-}events}$)
**pRNFL**									
All patients	1.243 (0.822-1.879)[a]	0.303*#	28.5% (116/407)	0.915 (0.618-1.354)	0.656	36.19% (135/373)	0.893 (0.621-1.285)	0.543*	49.02% (150/306)
All patients w/o ON	1.195 (0.702-2.035)[a]	0.512*	33.33% (76/228)	0.829 (0.52-1.322)	0.431	37.91% (105/277)	0.653 (0.407-1.046)	0.076*	48.42% (107/221)
RRMS	1.189 (0.742-1.906)	0.471*	48.92% (91/186)	0.926 (0.508-1.687)	0.801	36.41% (67/184)	0.889 (0.575-1.376)	0.599	60% (111/185)
RRMS w/o ON	1.201 (0.655-2.203)	0.553*	45.99% (63/137)	0.753 (0.365-1.554)	0.444	38.3% (54/141)	0.662 (0.386-1.138)	0.136	63.24% (86/136)
SPMS	1.422 (0.576-3.513)	0.446	41.82% (23/55)	0.93 (0.428-2.02)	0.854	36.25% (29/80)	0.682 (0.255-1.821)	0.445	35.19% (19/54)
SPMS w/o ON	1.271 (0.384-	0.695	40.62% (13/32)	0.742 (0.268-	0.566	39.53% (17/43)	0.31 (0.06-1.603)	0.162	22.58% (7/31)

	4.212)			2.053)					
PPMS	-	-	-	0.941 (0.446-1.988)	0.874	35.78% (39/109)	1.726 (0.656-4.543)	0.269	29.85% (20/67)
**mRNFL**									
All patients	0.863 (0.577-1.291)[a]	0.474*#	28.5% (116/407)	0.865 (0.602-1.243)	0.433*#	36.19% (135/373)	0.899 (0.631-1.281)	0.557*	49.02% (150/306)
All patients w/o ON	0.85 (0.5-1.445)[a]	0.547*	33.33% (76/228)	0.83 (0.543-1.268)	0.388	37.91% (105/277)	0.789 (0.502-1.239)	0.303*	48.42% (107/221)
RRMS	0.834 (0.511-1.362)	0.468*	48.92% (91/186)	0.865 (0.485-1.54)	0.622	36.41% (67/184)	0.963 (0.623-1.488)	0.864	60% (111/185)
RRMS w/o ON	0.739 (0.394-1.387)	0.346*	45.99% (63/137)	0.717 (0.357-1.441)	0.35	38.3% (54/141)	0.917 (0.544-1.545)	0.743	63.24% (86/136)
SPMS	1.227 (0.524-2.876)	0.637*	41.82% (23/55)	0.955 (0.452-2.016)	0.904	36.25% (29/80)	0.41 (0.164-1.021)	0.055	35.19% (19/54)
SPMS w/o ON	0.892 (0.286-2.783)	0.843	40.62% (13/32)	0.755 (0.282-2.019)	0.575	39.53% (17/43)	0.265 (0.055-1.264)	0.096	22.58% (7/31)
PPMS	-	-	-	0.64 (0.316-	0.214*	35.78% (39/109)	**2.814 (1.129-**	**0.026***	**29.85% (20/67)**

				1.293)			**7.013)**		
**GCIPL**									
All patients	0.955 (0.643-1.419)[a]	0.82*#	28.5% (116/407)	0.788 (0.542-1.145)	0.211*#	36.19% (135/373)	0.833 (0.579-1.197)	0.323*	49.02% (150/306)
All patients w/o ON	1.006 (0.607-1.667)[a]	0.981*	33.33% (76/228)	0.828 (0.542-1.264)	0.382	37.91% (105/277)	0.731 (0.463-1.155)	0.18*	48.42% (107/221)
RRMS	0.821 (0.504-1.34)	0.43*	48.92% (91/186)	0.707 (0.385-1.298)	0.263	36.41% (67/184)	0.773 (0.491-1.218)	0.267	60% (111/185)
RRMS w/o ON	0.881 (0.491-1.581)	0.672*	45.99% (63/137)	0.71 (0.363-1.39)	0.318	38.3% (54/141)	0.706 (0.414-1.204)	0.201	63.24% (86/136)
SPMS	1.742 (0.699-4.341)	0.234*	41.82% (23/55)	0.951 (0.443-2.044)	0.898	36.25% (29/80)	0.521 (0.211-1.286)	0.157	35.19% (19/54)
SPMS w/o ON	1.04 (0.332-3.265)	0.946	40.62% (13/32)	1.253 (0.471-3.335)	0.652	39.53% (17/43)	0.31 (0.06-1.612)	0.164	22.58% (7/31)
PPMS	-	-	-	0.724 (0.363-1.444)	0.359*	35.78% (39/109)	**2.607 (1.051-6.466)**	**0.039***	**29.85% (20/67)**
**INL**									
All	1.035	0.863*#	28.5%	0.831	0.317*#	36.19%	1.158	0.388*	49.02%

patients	(0.698-1.535)[a]		(116/407)	(0.579-1.194)		(135/373)	(0.83-1.614)		(150/306)
All patients w/o ON	1.149 (0.711-1.858)[a]	0.57*	33.33% (76/228)	0.889 (0.594-1.329)	0.565	37.91% (105/277)	1.45 (0.981-2.141)	0.062*	48.42% (107/221)
RRMS	1.164 (0.752-1.803)	0.495*	48.92% (91/186)	0.825 (0.494-1.377)	0.462	36.41% (67/184)	1.046 (0.707-1.545)	0.823	60% (111/185)
RRMS w/o ON	1.285 (0.763-2.162)	0.346*	45.99% (63/137)	0.953 (0.543-1.671)	0.865	38.3% (54/141)	1.214 (0.778-1.896)	0.393	63.24% (86/136)
SPMS	0.897 (0.373-2.158)	0.808*	41.82% (23/55)	0.781 (0.36-1.693)	0.531	36.25% (29/80)	0.741 (0.281-1.954)	0.544	35.19% (19/54)
SPMS w/o ON	0.744 (0.224-2.474)	0.629	40.62% (13/32)	0.854 (0.313-2.33)	0.758	39.53% (17/43)	0.651 (0.125-3.387)	0.61	22.58% (7/31)
PPMS	-	-	-	0.494 (0.239-1.018)	0.056	35.78% (39/109)	**2.526 (1.028-6.207)**	**0.043***	**29.85% (20/67)**

Legend: Presented hazard ratios of retinal layer thickness represent the hazard ratio of having a thickness in the lowest tertile compared to the upper two. The proportion of patients experiencing relapses, disability worsening (EDSS progression), and MRI progression/activity is displayed for baseline pRNFL, mRNFL, GCIPL, and INL thickness. Thickness tertiles were defined as pRNFL ≤ 88 µm, mRNFL ≤ 29 µm, GCIPL ≤ 62 µm, NL ≤ 33 µm. Results are presented for all patients (RRMS, SPMS, and PPMS), for all patients (RRMS, SPMS) without previous ON, for all RRMS and those without previous ON, all SPMS and those without previous ON, and all PPMS. Without previous ON means without ON based on medical history and without ON based on inter-eye GCIPL thickness difference [43]. [a] excluding patients with PPMS. w/o = without. The following covariates were included in the analysis: *Age; #Sex. All statistical tests were two-sided. Source data are provided as a Source Data file.

Supplementary Table 10 Overview of high- and low-efficacy DMTs at baseline

**Low-efficacy DMTs**
Interferon beta
Glatiramer acetate
Teriflunomide
Dimethyl fumarate
**High-efficacy DMTs**
Ocrelizumab
Rituximab
Mitoxantrone
Fingolimod
Cyclophosphamide
Natalizumab
Alemtuzumab
Daclizumab
Cladribine
Treosulfan
Hematopoietic stem cell transplantation
Immunoglobulins
Mycophenolate mofetil

Supplementary Figure 1 Spline regressions between retinal layer thickness, visual acuity, and VEP latency at baseline and disease duration.

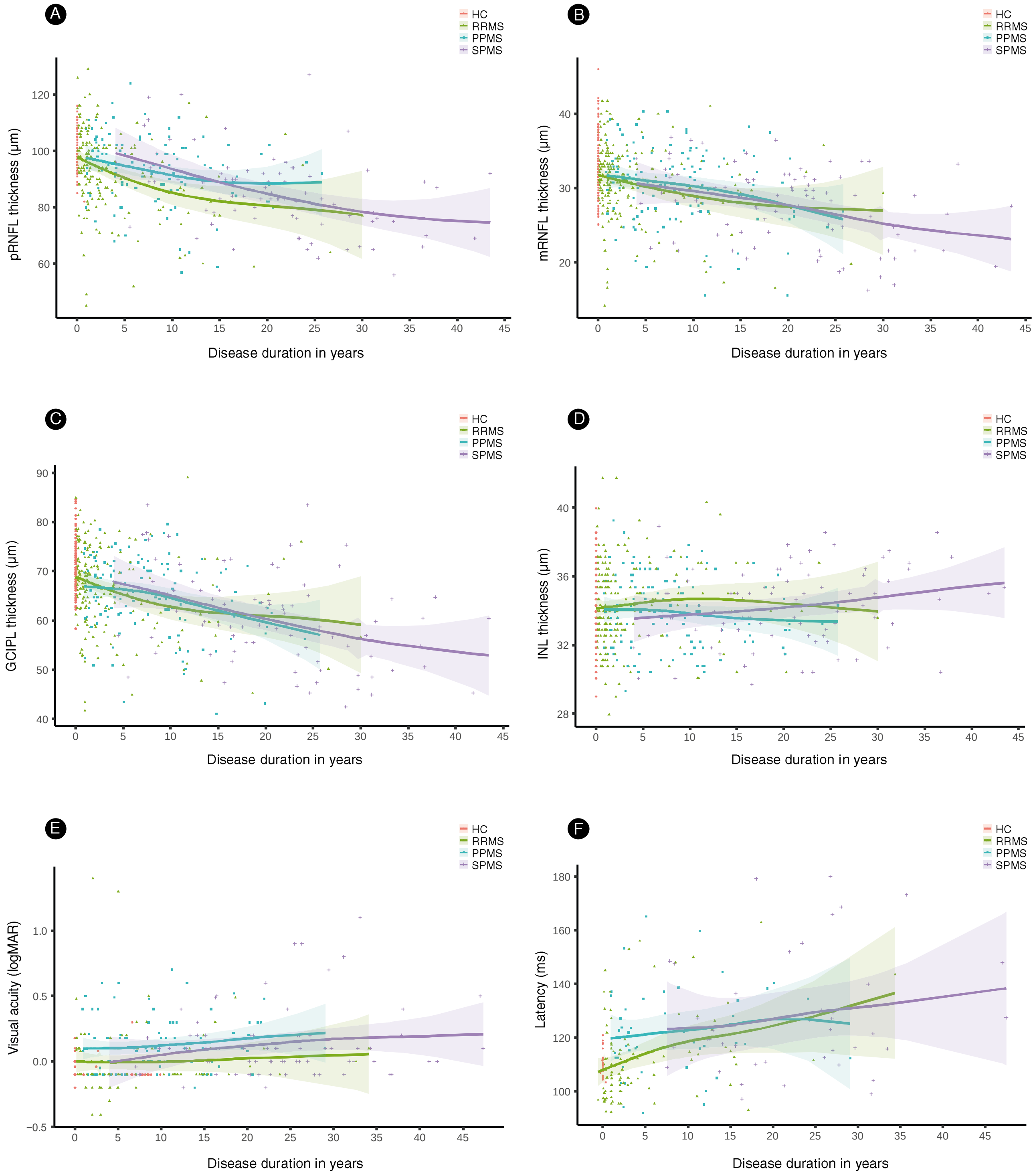

The raw data of retinal layer thickness (A: pRNFL, B: mRNFL, C: GCIPL; D: INL), visual acuity (E), and latency (F) are plotted against the disease duration at baseline, each dot representing a single eye. 95% CI based on jackknife residuals. Splines were fitted with three degrees of freedom. Source data are provided as a Source Data file.

Supplementary Figure 2 Spline regressions between retinal layer thickness, visual acuity, and VEP latency at baseline and age.

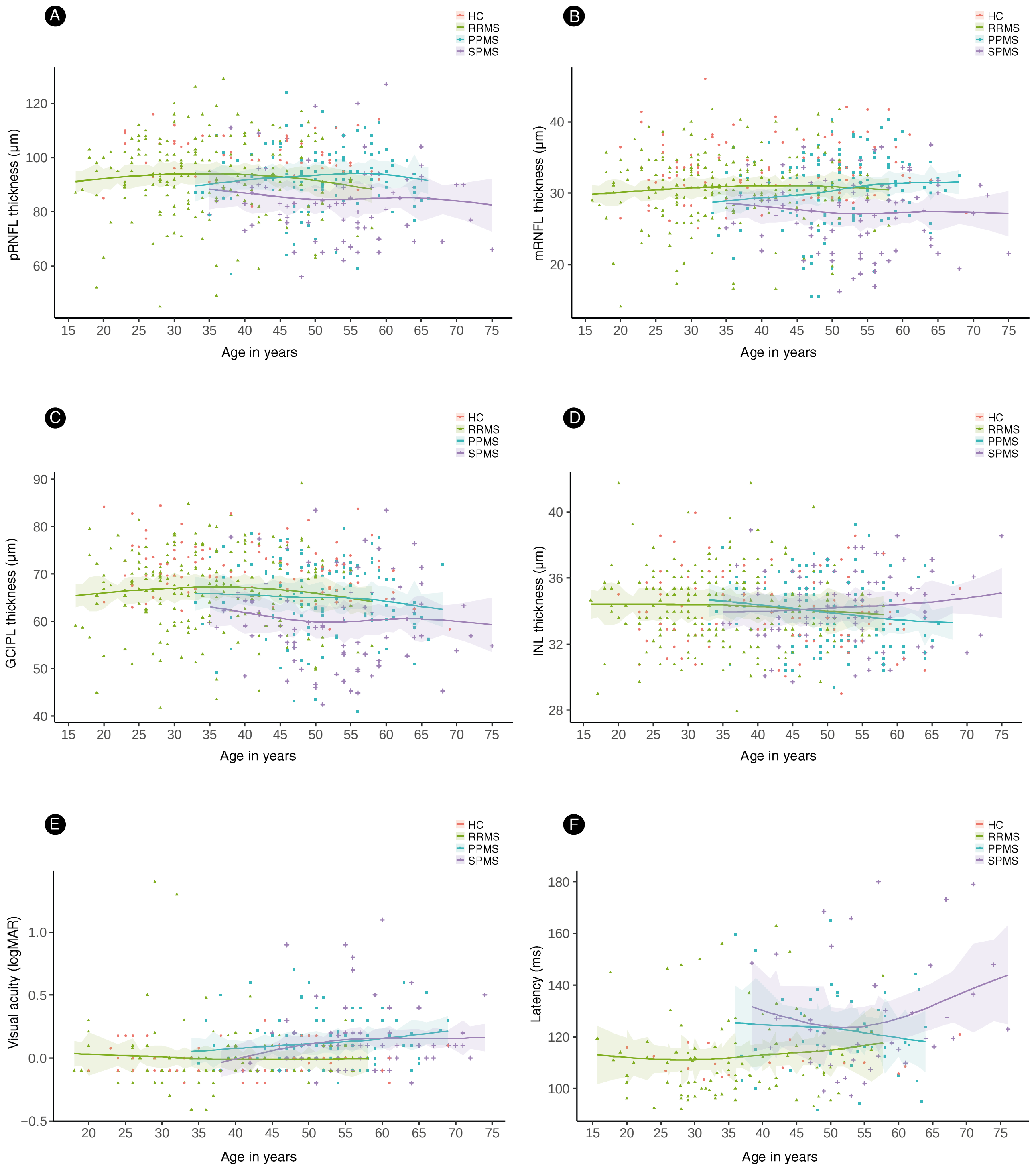

The raw data of retinal layer thickness (A: pRNFL, B: mRNFL, C: GCIPL; D: INL), visual acuity (E), and latency (F) are plotted against age at baseline, each dot representing a single eye. 95% CI based on jackknife residuals. Splines were fitted with three degrees of freedom. Source data are provided as a Source Data file.

Supplementary Figure 3 Spline regressions of retinal layer thickness, visual acuity, and VEP latency plotted against age over time.

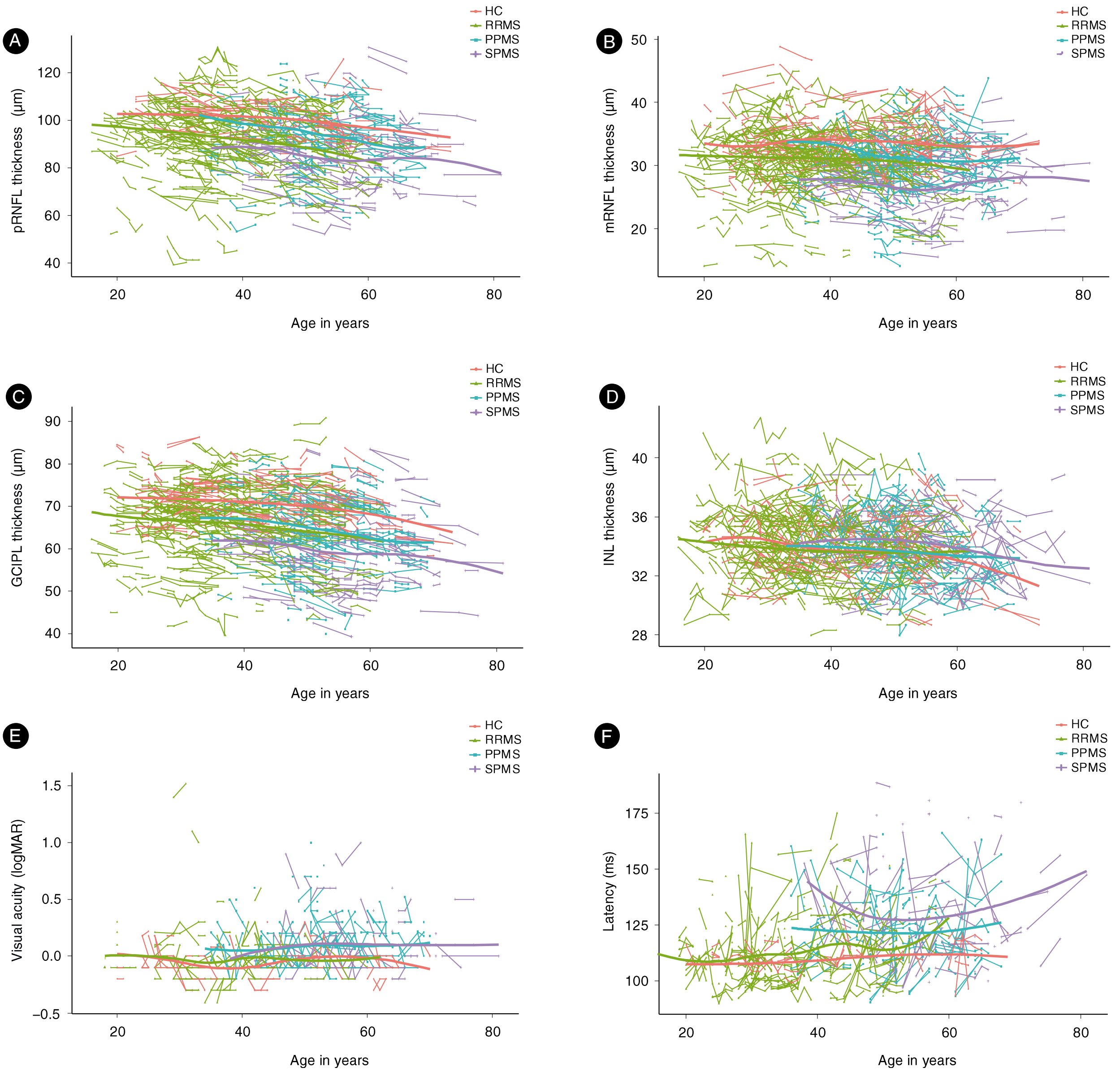

The raw data of retinal layer thickness (A: pRNFL, B: mRNFL, C: GCIPL; D: INL), visual acuity (E), and latency (F) are plotted over age, each dot representing a single eye and follow-up assessments being connected with lines. 95% CI based on jackknife residuals. B-splines were fitted in a mixed model with random intercept for eyes and controlling for gender. The number of knots and polynominal degrees (both between 1 and 5) were chosen such that 10-fold cross validation mean squared error (MSE) was minimized. Source data are provided as a Source Data file.

Supplementary Figure 4 Empirical Bayes estimates of best linear unbiased predictions (BLUPs) of thickness change rate, reporting histograms of estimates across disease courses.

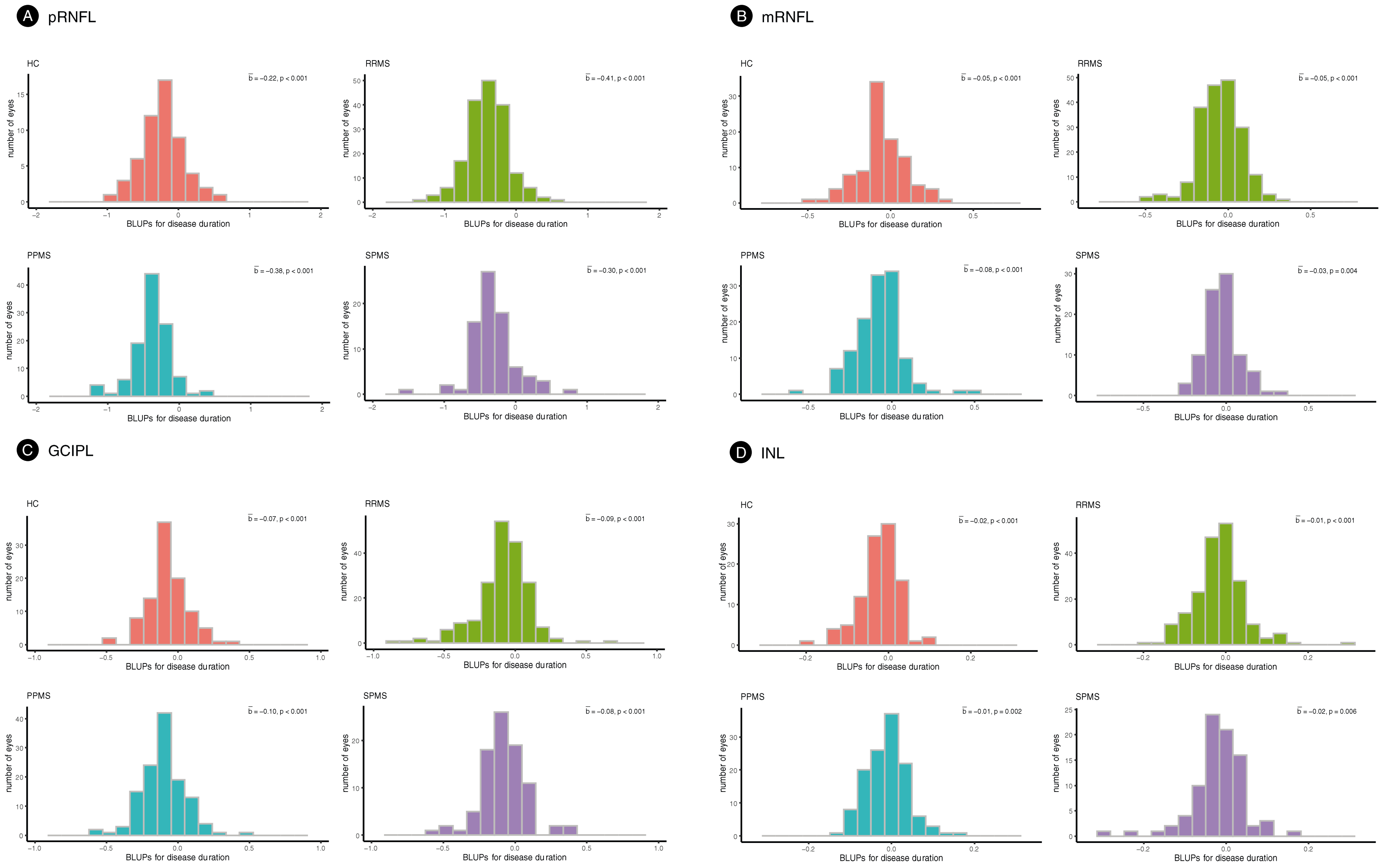

BLUPs demonstrated significant atrophy of all retinal layers (A: pRNFL, B: mRNFL, C: GCIPL; D: INL) for all groups (HC, RRMS, PPMS, SPMS). Source data are provided as a Source Data file.

Supplementary Figure 5
Results of the eye wise ordinary least squares (OLS) with disease duration as only predictor and aggregrated regression coefficients.

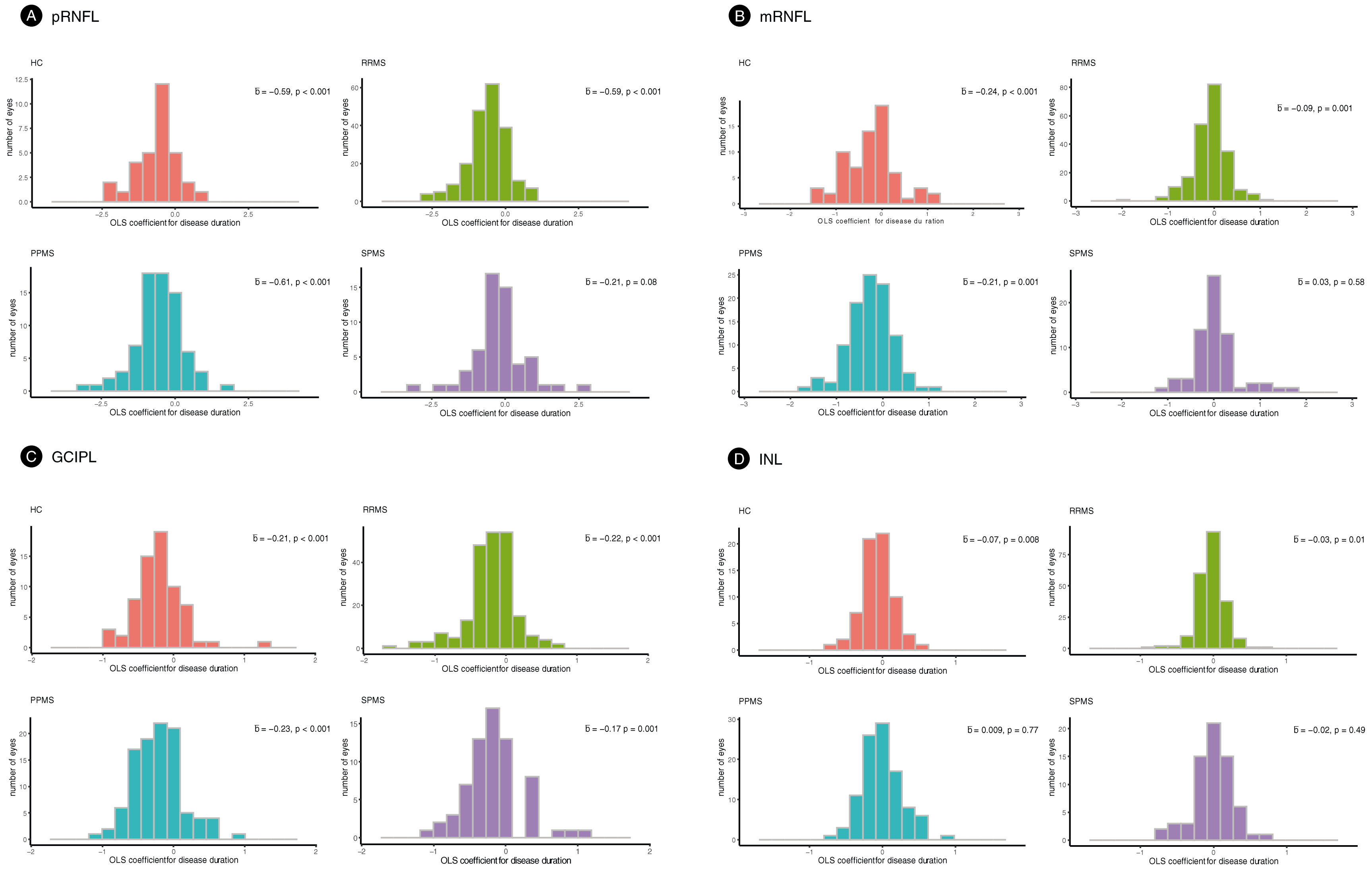

Coefficients were estimated over the entire disease duration. Eyes with less than three OCTs were discarded. Wilcoxon rank-sum tests demonstrated significant atrophy of all retinal layers of HCs, RRMS, SPMS, and PPMS patients, except for SPMS with regard to pRNFL, mRNFL, and INL and PPMS with regard to INL. Source data are provided as a Source Data file.
