# Supplementary material for: Evolution of retinal degeneration and prediction of disease activity in relapsing and progressive multiple sclerosis
Source: Nat Commun. 2024 Jun 19;15:5243. doi: 10.1038/s41467-024-49309-7 (PMC11187157; doi:10.1038/s41467-024-49309-7)
Supplement: Supplementary file 3 — Reporting Summary [file 41467_2024_49309_MOESM3_ESM.pdf]

nature portfolio

Corresponding author(s): Julia Krämer and Philipp Albrecht

Last updated by author(s): Mar 29, 2024

Reporting Summary

Nature Portfolio wishes to improve the reproducibility of the work that we publish. This form provides structure for consistency and transparency in reporting. For further information on Nature Portfolio policies, see our Editorial Policies and the Editorial Policy Checklist.

Statistics

For all statistical analyses, confirm that the following items are present in the figure legend, table legend, main text, or Methods section.

n/a	Confirmed	
☐	☒	The exact sample size (*n*) for each experimental group/condition, given as a discrete number and unit of measurement
☐	☒	A statement on whether measurements were taken from distinct samples or whether the same sample was measured repeatedly
☐	☒	The statistical test(s) used AND whether they are one- or two-sided<br>*Only common tests should be described solely by name; describe more complex techniques in the Methods section.*
☐	☒	A description of all covariates tested
☐	☒	A description of any assumptions or corrections, such as tests of normality and adjustment for multiple comparisons
☐	☒	A full description of the statistical parameters including central tendency (e.g. means) or other basic estimates (e.g. regression coefficient) AND variation (e.g. standard deviation) or associated estimates of uncertainty (e.g. confidence intervals)
☐	☒	For null hypothesis testing, the test statistic (e.g. *F*, *t*, *r*) with confidence intervals, effect sizes, degrees of freedom and *P* value noted<br>*Give P values as exact values whenever suitable.*
☒	☐	For Bayesian analysis, information on the choice of priors and Markov chain Monte Carlo settings
☐	☒	For hierarchical and complex designs, identification of the appropriate level for tests and full reporting of outcomes
☐	☒	Estimates of effect sizes (e.g. Cohen's *d*, Pearson's *r*), indicating how they were calculated

Our web collection on statistics for biologists contains articles on many of the points above.

Software and code

Policy information about availability of computer code

Data collection	No software was used
Data analysis	SPSS Statistics 26.0 (IBM) or the lme4 package in R Studio (version 1.3.1093) and Microsoft Excel 2021. All statistical codes for presented results are available under: https://github.com/AlexHartmann00/oct-ms.

For manuscripts utilizing custom algorithms or software that are central to the research but not yet described in published literature, software must be made available to editors and reviewers. We strongly encourage code deposition in a community repository (e.g. GitHub). See the Nature Portfolio guidelines for submitting code & software for further information.

Data

Policy information about availability of data

All manuscripts must include a data availability statement. This statement should provide the following information, where applicable:

- Accession codes, unique identifiers, or web links for publicly available datasets
- A description of any restrictions on data availability
- For clinical datasets or third party data, please ensure that the statement adheres to our policy

The datasets generated during and/ or analysed during the current study are available from the corresponding authors (J.K. or P.A) on reasonable request.

Human research participants

Policy information about studies involving human research participants and Sex and Gender in Research.

Reporting on sex and gender	According to the Nature guidelines, we have used the term sex to describe the biological attribute. We considered both sexes. Sex was assigned according to hospital recordings. 241 of 407 MS patients were female. It is described in the source data where the information of sex has been collected. Consent has been obtained for sharing of individual-level data of all participants. All analyses were corrected for participants' sex.
Population characteristics	Datasets of patients with RRMS (N=261), SPMS (N=139), and PPMS (N=190) who were diagnosed according to the revised McDonald criteria 2017 were obtained from five MS centers (Düsseldorf, Münster, Berlin, Hamburg, and Munich). 47.7% of patients were female. The median age and disease duration of all patients at baseline were 45 and 5 years respectively.
Recruitment	Patients had been recruited in the context of different non-interventional longitudinal OCT studies at five MS centers (Düsseldorf, Münster, Berlin, Hamburg, and Munich) from 13.10.2009 to 18.05.2020.
Ethics oversight	Patients and healthy controls participated in observational studies which were approved by the local ethics committees (Düsseldorf: 5794R and 4389R, Münster: 2017-754-f-S, Berlin: EA1/182/10 and EA1/163/12, Hamburg: PV4455, PV3961 and PV5557, Munich: 427-14), and provided written informed consent for participation.

Note that full information on the approval of the study protocol must also be provided in the manuscript.

Field-specific reporting

Please select the one below that is the best fit for your research. If you are not sure, read the appropriate sections before making your selection.

[x] Life sciences [ ] Behavioural & social sciences [ ] Ecological, evolutionary & environmental sciences

For a reference copy of the document with all sections, see nature.com/documents/nr-reporting-summary-flat.pdf

Life sciences study design

All studies must disclose on these points even when the disclosure is negative.

Sample size	This was a retrospective study involving all available datasets from the participating centers. Due to the retrospective nature of the study, no power analysis was performed and due to the exploratory nature sensitivits analyses were not performed.
Data exclusions	Exclusion criteria were any diseases of the optic nerve or retina not related to MS; a diagnosis of other neuroinflammatory disorders (i.e., neuromyelitis optica spectrum disorders); severe refraction anomalies ≥ ± 6 diopters; systemic conditions that could affect the visual system; treatment with substances with increased risk of iatrogenic retinopathy such as chemotherapy; insufficient scan quality according to the OSCAR-IB criteria. In MS patients, initial swelling and retinal atrophy in the context of acute ON has a major impact on retinal layer thickness. For this reason, we excluded eyes with previous ON within 6 months to baseline OCT and those with ON between OCT measurements. Exclusion criteria were predefined.
Replication	Reproduction in an independent cohort will be of interest but was beyond the scope of this study. Nevertheless some of our results have been found in other studies and can thus be considered as reproduced.
Randomization	non applicable, retrospective study
Blinding	Investigators were blinded for group allocation

Reporting for specific materials, systems and methods

We require information from authors about some types of materials, experimental systems and methods used in many studies. Here, indicate whether each material, system or method listed is relevant to your study. If you are not sure if a list item applies to your research, read the appropriate section before selecting a response.

Materials & experimental systems

n/a	Involved in the study
☒	☐ Antibodies
☒	☐ Eukaryotic cell lines
☒	☐ Palaeontology and archaeology
☒	☐ Animals and other organisms
☒	☐ Clinical data
☒	☐ Dual use research of concern

Methods

n/a	Involved in the study
☒	☐ ChIP-seq
☒	☐ Flow cytometry
☒	☐ MRI-based neuroimaging
